# Fear of missing out (FoMO) in the digital age: implications for academic productivity and sleep quality among Saudi university students

**DOI:** 10.3389/fpsyg.2026.1847930

**Published:** 2026-05-28

**Authors:** Fahad Alzahrani, Raid Raidan, Wejdan Al Harbi, Sadeem S. Aljutily, Leen Almuzainy, Jood Aljohani, Enas Aljohani, Majed Alrefaei, Salman Aljohani, Yousef Alorfi, Sultan S. Al Thaqfan, Saad B. Fallatah, Sultan Othman Alolayan, Ehsan Habeeb, Faris S. Alnezary

**Affiliations:** 1 Department of Pharmacy Practice, College of Pharmacy, Taibah University, Madinah, Saudi Arabia; 2 Scientific Research Unit, College of Pharmacy, Taibah University, Madinah, Saudi Arabia

**Keywords:** academic productivity, fear of missing out, Saudi Arabia, sleep quality, social media

## Abstract

**Background:**

Fear of missing out (FoMO) has emerged as an important psychological concern among university students, associated with excessive social media engagement and linked to academic and sleep-related outcomes. However, evidence from Middle Eastern populations remains limited. Therefore, this study aimed to examine the relationships among FoMO, problematic social media use, academic productivity, and sleep quality among university students in Saudi Arabia.

**Methods:**

A cross-sectional study was conducted among university students in Saudi Arabia using a self-administered online questionnaire. FoMO was assessed using an adapted FoMO scale, problematic social media use using validated social media addiction items, academic productivity using validated academic behavior items, and sleep quality using the Pittsburgh Sleep Quality Index (PSQI). Pearson correlation and multiple linear regression analyses were performed.

**Results:**

A total of 1,071 participants were included. FoMO was positively correlated with problematic social media use (*r* = 0.55, *p* < 0.01) and poorer sleep quality (*r* = 0.25, *p* < 0.01). Problematic social media use was negatively associated with academic productivity (*r* = −0.11, *p* < 0.01) and positively associated with poorer sleep quality (*r* = 0.20, *p* < 0.01). Regression analysis showed that problematic social media use was negatively associated with academic productivity (*β* = −0.17, *p* < 0.001), while FoMO (*β* = 0.19, *p* < 0.001) and problematic social media use (*β* = 0.11, *p* = 0.002) were positively associated with poorer sleep quality.

**Conclusion:**

FoMO was associated with problematic social media use and poorer sleep quality, while problematic social media use was negatively associated with academic productivity among university students. These findings highlight the importance of promoting healthy digital behaviors to support student well-being and academic outcomes.

## Introduction

The rapid expansion of social media has transformed communication, information access, and social interaction among university students. Although social media offers opportunities for academic collaboration and connectivity, excessive engagement has also been associated with psychological and behavioral concerns, particularly fear of missing out (FoMO) (Yao et al., 2025; Kohanová et al., 2025). FoMO is defined as a pervasive apprehension that others may be experiencing rewarding events from which one is absent, accompanied by a strong desire to remain continuously connected to social networks (Groenestein et al., 2024). Previous research has linked FoMO to compulsive social media use, emotional distress, and maladaptive behavioral patterns that may negatively affect daily functioning and well-being (Lust and Danzey, 2025; Huang et al., 2023; Jing et al., 2025; Zhu et al., 2023).

Self-Determination Theory (SDT) provides a useful framework for understanding these relationships. SDT proposes that individuals are motivated by the fulfillment of autonomy, competence, and relatedness needs (Przybylski et al., 2013). When these needs are unmet, individuals may seek social validation and connectedness through digital environments. Within this framework, FoMO may reflect unmet needs for relatedness, leading individuals to engage excessively with social media to avoid perceived social exclusion (Lust and Danzey, 2025). Consequently, problematic social media use may emerge as a maladaptive coping strategy that can negatively influence academic productivity and sleep quality (West et al., 2024; Alhusban et al., 2022; Khalaf et al., 2025).

The relationship between FoMO and problematic social media use is particularly relevant among university students, who represent one of the most active groups of social media users (Zhu et al., 2023; Elhadary and Abdelatti, 2024). Existing studies suggest that problematic social media use is associated with reduced concentration, procrastination, and poorer academic performance (Zhu et al., 2023; Kolhar et al., 2021). Similarly, excessive social media engagement has been associated with delayed sleep onset and poorer sleep quality, although previous findings differ regarding whether these effects arise primarily from screen exposure or psychological mechanisms such as FoMO and emotional arousal (Scott and Woods, 2018; Almeida et al., 2023; Scott et al., 2019). These inconsistencies suggest that FoMO may represent an important psychological pathway linking problematic social media use with academic and sleep-related outcomes.

Despite growing research, several gaps remain. First, most evidence originates from Western populations, limiting generalizability to Middle Eastern contexts, particularly Saudi Arabia, where social media use is highly prevalent (Przybylski et al., 2013; Abd Ellatif Elsayed, 2025). Second, prior studies have often examined FoMO, social media use, academic productivity, and sleep quality separately rather than within an integrated theoretical framework (Almeida et al., 2023; Abd Ellatif Elsayed, 2025). Third, inconsistent findings regarding academic productivity indicate that additional psychological and behavioral mechanisms may influence these relationships (Gong et al., 2025; Lin et al., 2024).

The present study aimed to investigate the associations among FoMO, problematic social media use, academic productivity, and sleep quality among university students in Saudi Arabia using Self-Determination Theory as a guiding framework. Based on previous literature, a conceptual model was developed to illustrate the hypothesized relationships among FoMO, problematic social media use, academic productivity, and sleep quality. The following hypotheses were proposed: H1, FoMO is positively associated with problematic social media use; H2, problematic social media use is negatively associated with academic productivity; H3, FoMO is positively associated with poorer sleep quality; and H4, problematic social media use is positively associated with poorer sleep quality. In addition, problematic social media use was conceptually examined as a behavioral factor associated with the relationships between FoMO, academic productivity, and poorer sleep quality.

## Methods

### Study design and participants

A cross-sectional study was conducted among university students in Saudi Arabia to examine the relationship between fear of missing out (FoMO), social media use, academic productivity, and sleep quality. Data were collected using a self-administered online questionnaire distributed through university communication channels and social media platforms.

A non-probability sampling approach was employed, combining convenience and snowball sampling techniques. The survey link was initially shared through university groups and digital platforms, and participants were encouraged to distribute it within their academic networks to increase reach. The required sample size was estimated using a 95% confidence level, 5% margin of error, and an assumed population proportion of 50%. Because no prior prevalence estimates relevant to the study population were available, a conservative 50% proportion was selected to maximize the required sample size and ensure adequate statistical power. Based on this calculation, the minimum required sample size was 384 participants.

Eligibility criteria included being an undergraduate student currently enrolled in a university in Saudi Arabia and aged 18 years or older. Participants up to age 30 were eligible because undergraduate enrollment in Saudi universities may include mature students, delayed academic progression, bridging programs, and individuals returning to complete their studies through non-traditional educational pathways. Participants who did not complete the questionnaire were excluded from the analysis. The sample consisted of a higher proportion of female students compared to males. This distribution is consistent with national higher education statistics in Saudi Arabia, where female students represent a larger share of university enrollment (Elhadary and Abdelatti, 2024). Therefore, the observed gender distribution in this study reflects the underlying population structure.

### Data collection procedure

Data were collected using a structured, self-administered online questionnaire distributed through digital platforms. The survey was developed using an online survey tool and disseminated via university communication channels and social media platforms, including student groups and messaging applications, to maximize reach among undergraduate students. Prior to participation, all students were presented with an electronic informed consent form outlining the purpose of the study, the procedures, the voluntary nature of participation, and the confidentiality of responses. Participants were required to provide consent before proceeding with the questionnaire. No personally identifiable information was collected, ensuring anonymity and reducing the risk of response bias. The survey’s anonymity and voluntariness were also intended to reduce the potential influence of common method bias associated with self-reported measures.

The questionnaire was designed in a sequential format. It began with demographic questions, followed by sections assessing fear of missing out (FoMO), problematic social media use, academic productivity, and sleep quality. Clear instructions were provided at the beginning of each section to guide participants in responding based on their personal experiences. The survey utilized Likert-type response scales to ensure consistency across measures. To enhance clarity and content validity, the questionnaire was reviewed prior to distribution to ensure that all items were understandable and appropriately structured. Participants could complete the survey at their convenience, and responses were automatically recorded and securely stored within the survey platform. As an incentive to encourage participation, respondents were given the option to enter a prize draw after completing the survey. Three participants were randomly selected to receive a monetary reward of 200 Saudi Riyals each. Participation in the prize draw was optional and did not affect participation in the study.

### Measures

The survey instrument consisted of multiple sections assessing demographic characteristics, fear of missing out (FoMO), problematic social media use, academic productivity, and sleep quality. All items were self-reported and measured using Likert-type scales. Composite mean scores were calculated for each construct by averaging responses across related items, with higher scores indicating greater levels of the measured construct. Aggregation of items was justified on the basis of conceptual similarity and acceptable internal consistency reliability coefficients. All scales used 5-point response formats, and composite mean scores ranged from 1 to 5.

#### Demographic characteristics

Participants provided information on age, gender, university, field of study, academic year, grade point average (GPA), daily social media use, study hours, living arrangement, and monthly allowance. These variables were used to describe the sample and to examine differences in FoMO across subgroups.

#### Fear of missing out (FoMO)

FoMO was assessed using a multi-item scale adapted from the original FoMO scale developed by Przybylski et al. (2013). The scale measures individuals’ concerns about missing rewarding social, academic, and personal experiences. The construct consisted of multiple items assessing emotional concern about exclusion and the desire to remain socially connected. Example items included “I fear my friends have more rewarding experiences than me” and “I feel anxious when I do not know what my friends are up to.” Responses were recorded on a 5-point Likert scale, and a composite mean score was calculated, ranging from 1 to 5, with higher scores indicating greater levels of FoMO. The scale demonstrated good internal consistency (Cronbach’s *α* = 0.86).

#### Problematic social media use

Problematic social media use was assessed using platform-specific items adapted from prior research on social media addiction (Andreassen et al., 2016; Bányai et al., 2017; Boer et al., 2020). The scale evaluated dimensions of compulsive use, emotional dependence, loss of control, and interference with daily functioning across commonly used social media platforms. Example items included “I find it difficult to reduce my social media use” and “Social media use interferes with my academic and personal responsibilities.” Responses were recorded on a 5-point Likert scale, and a composite mean score ranging from 1 to 5 was calculated, with higher scores indicating greater problematic social media use. The scale demonstrated good internal consistency in the present study (Cronbach’s *α* = 0.87).

#### Academic productivity

Academic productivity was assessed using items evaluating students’ study behaviors, time management, and academic engagement (Dumrique and Castillo, 2018; Hysenbegasi et al., 2005). The construct consisted of multiple items reflecting perceived effectiveness in completing academic tasks and maintaining consistent study routines. Example items included “I manage my study time effectively” and “I am able to concentrate during academic activities even when I receive social media notifications.” Responses were recorded on a 5-point Likert scale, and a composite mean score ranging from 1 to 5 was calculated, with higher scores indicating better academic productivity. The scale demonstrated good internal consistency in the present study (Cronbach’s *α* = 0.86).

#### Sleep quality

Sleep quality was assessed using the Pittsburgh Sleep Quality Index (PSQI), a validated instrument designed to evaluate sleep patterns and disturbances over the previous month (Buysse et al., 1989). The scale included items assessing subjective sleep quality, sleep duration, sleep latency, and difficulties initiating sleep. Example items included “How often have you felt that your overall sleep quality was poor or unsatisfying?” and “How often have you had trouble sleeping because you could not fall asleep within 30 minutes?” Responses were recorded on a 5-point scale, and a composite mean score ranging from 1 to 5 was calculated, with higher scores indicating poorer sleep quality and greater sleep-related difficulties. The scale demonstrated acceptable internal consistency in the present study (Cronbach’s *α* = 0.78).

### Face validity, pilot testing, and reliability

Content validity of the study tool was established through expert review by three researchers with experience in academic and health-related research. Prior to data collection, all items were evaluated for clarity, relevance, and appropriateness within the study context. The tool was pilot tested with 25 participants to assess clarity and readability. Reliability was evaluated using the test–retest method, whereby participants completed the questionnaire twice within a short interval (30 min to 1 h). The results demonstrated excellent stability (*r* = 0.95, 95% CI: 0.91–0.99; *p* < 0.001). However, the short retest interval may have increased the risk of recall bias and may not fully reflect temporal stability. Therefore, this issue has been acknowledged as a methodological limitation in the revised manuscript.

### Statistical analysis

Data were analyzed using parametric statistical methods. Normality was assessed using histograms, Q–Q plots, and skewness and kurtosis values. Descriptive statistics, including frequencies, percentages, means, and standard deviations, were used to summarize participant characteristics and study variables. Independent-samples *t*-tests were used for binary variables, whereas one-way analysis of variance (ANOVA) was used for variables with three or more categories. Significant ANOVA findings were followed by Tukey’s honestly significant difference (HSD) *post hoc* analysis. Cases with significant overall associations but non-significant pairwise comparisons were interpreted cautiously. Pearson correlation coefficients were calculated to examine associations among FoMO, problematic social media use, academic productivity, and poorer sleep quality. Spearman correlation analysis was conducted as a sensitivity analysis and produced comparable findings.

Multiple linear regression analyses were performed to examine associations between variables and academic productivity and poorer sleep quality. Independent variables included FoMO, problematic social media use, gender, age, GPA, study hours, and daily social media use. GPA was treated as an indicator associated with academic performance rather than as a causal predictor. Problematic social media use and daily social media use were included because they represent different dimensions of social media behavior. Daily social media use reflects duration of use, whereas problematic social media use reflects maladaptive and compulsive engagement patterns. Regression assumptions were evaluated prior to analysis. Residual plots indicated acceptable normality and homoscedasticity. Multicollinearity diagnostics showed no evidence of problematic collinearity, with VIF values ranging from 1.01 to 1.56 and tolerance values ranging from 0.64 to 0.99. Regression coefficients were reported with 95% confidence intervals. Statistical significance was set at *p* < 0.05.

### Ethical considerations

The study was conducted in accordance with the ethical principles outlined in the Declaration of Helsinki. Ethical approval was obtained from the Scientific Research Ethics Committee of the College of Pharmacy at Taibah University, Saudi Arabia (Reference No: COPTU-REC-155-20250915). Participation in the study was voluntary, and all participants provided informed consent prior to completing the survey. Participants were informed about the study objectives, procedures, and their right to withdraw at any time without any consequences. To ensure confidentiality and privacy, no personally identifiable information was collected, and all responses were recorded anonymously.

## Results

### Student characteristics and FOMO scores

The majority of participants were female (76.0%) and aged 18–20 years (58.1%). Most students were from Western universities (55.3%), and a large proportion were enrolled in health (40.5%) and scientific disciplines (36.0%).

Overall, FoMO scores showed no statistically significant differences across gender, age groups, university location, academic year, GPA, or average daily study hours (all *p* > 0.05). However, Significant differences in FOMO scores were observed across academic disciplines (*p* = 0.02). *Post hoc* analysis using Tukey’s test indicated that students in health colleges reported significantly higher FoMO scores compared to those in administrative and humanitarian colleges (*p* = 0.015), while no significant differences were observed between health and scientific colleges or between scientific and administrative/humanitarian colleges. Living arrangement was significantly associated with FoMO (*p* = 0.03). However, *post hoc* pairwise comparisons did not reveal statistically significant differences between specific groups. Therefore, these findings should be interpreted cautiously despite the significant overall association.

In addition, daily social media use was significantly associated with FOMO (*p* = 0.02). Post hoc analysis using Tukey’s test showed that participants who used social media for 5–6 h per day had significantly higher FoMO scores compared to those who used it for 3–4 h (*p* = 0.024). No other pairwise comparisons were statistically significant, although a general trend toward higher FoMO scores was observed with increasing duration of social media use (Table 1).

**Table 1:** Students’ demographic characteristics and differences in FOMO scores.

Characteristics	Category	*n*	%	FOMO (M ± SD)	*p*-value
Gender					
Male		257	24.0	2.34 ± 0.78	0.12
Female		814	76.0	2.24 ± 0.72	
Age					
18–20		622	58.1	2.24 ± 0.72	0.32
21–23		379	35.4	2.31 ± 0.75	
24–30		70	6.5	2.32 ± 0.79	
University location					
Central		173	16.2	2.27 ± 0.77	0.45
Western		592	55.3	2.27 ± 0.71	
Eastern		122	11.4	2.34 ± 0.74	
Southern		62	5.8	2.30 ± 0.75	
Northern		122	11.4	2.17 ± 0.77	
Living arrangement					
With family		916	85.5	2.24 ± 0.73	0.03^*,^^a^
On campus		62	5.8	2.43 ± 0.71	
Alone/with roommates		93	8.7	2.39 ± 0.77	
Academic discipline					
Health		434	40.5	2.33 ± 0.75	0.02^*^
Scientific		386	36.0	2.26 ± 0.74	
Administrative/humanities		251	23.4	2.17 ± 0.69	
Academic year					
First year		258	24.1	2.20 ± 0.71	0.11
Second year		224	20.9	2.33 ± 0.77	
Third year		267	24.9	2.22 ± 0.71	
Fourth year		192	17.9	2.34 ± 0.71	
Fifth year		67	6.3	2.20 ± 0.74	
≥Sixth year		63	5.9	2.39 ± 0.84	
GPA (out of 5)					
<2.5		10	0.9	2.06 ± 0.60	0.49
2.5–3.0		58	5.4	2.13 ± 0.80	
3.1–3.5		122	11.4	2.25 ± 0.73	
3.6–4.0		207	19.3	2.27 ± 0.78	
>4.0		674	62.9	2.29 ± 0.72	
Daily study hours					
<1 h		151	14.1	2.21 ± 0.98	0.85
1–2 h		291	27.2	2.24 ± 0.72	
3–4 h		355	33.1	2.17 ± 0.69	
>4 h		274	25.6	2.34 ± 0.69	
Daily social media use					
<1 h		28	2.6	2.21 ± 0.98	0.02^*^
1–2 h		125	11.7	2.24 ± 0.72	
3–4 h		337	31.5	2.17 ± 0.69	
5–6 h		312	29.1	2.34 ± 0.69	
>6 h		269	25.1	2.33 ± 0.79	

a Significant overall ANOVA result; however, *post hoc* pairwise comparisons were not statistically significant and should therefore be interpreted cautiously. ^*^p < 0.05.

### Distribution of most frequently used social media platforms

**Figure 1:**
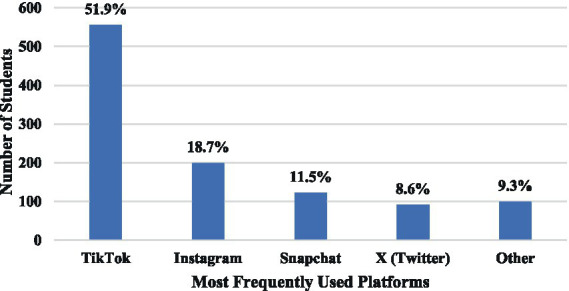
Distribution of the most frequently used social media platforms among participants.

The majority of participants reported TikTok as their most frequently used platform (51.9%), followed by Instagram (18.7%), Snapchat (11.5%), other platforms (9.3%), and X (Twitter) (8.6%) (Figure 1).

### Descriptive statistics and reliability

Overall, participants reported moderate levels of fear of missing out (FoMO) (mean = 2.26 ± 0.73) and relatively lower levels of problematic social media use (mean = 1.94 ± 0.78). Academic productivity scores were comparatively higher (mean = 2.96 ± 0.86), while sleep quality scores indicated poorer sleep patterns (mean = 3.32 ± 0.80). The measures demonstrated acceptable to high internal consistency, with Cronbach’s alpha coefficients ranging from 0.78 to 0.87 (Table 2).

**Table 2:** Mean scores and reliability of FoMO, social media usage, productivity, and sleep quality.

Variable	Mean ± SD	Cronbach’s *α*
FoMO	2.26 ± 0.73	0.86
Problematic social media use	1.94 ± 0.78	0.87
Academic productivity	2.96 ± 0.86	0.86
Sleep quality	3.32 ± 0.80	0.78

### Correlation analysis of study variables

Pearson correlation analysis showed that FoMO was positively correlated with problematic social media use (*r* = 0.55, *p* < 0.01) and poorer sleep quality (*r* = 0.25, *p* < 0.01), and weakly positively correlated with academic productivity (*r* = 0.07, *p* < 0.05). Problematic social media use was negatively associated with academic productivity (*r* = −0.11, *p* < 0.01) and positively associated with poorer sleep quality (*r* = 0.20, *p* < 0.01). Academic productivity was also positively correlated with poorer sleep quality (*r* = 0.24, *p* < 0.01), although this association was relatively weak and should therefore be interpreted cautiously. Age showed very weak associations with the study variables. It was positively correlated with problematic social media use (*r* = 0.08, *p* < 0.01) and negatively correlated with academic productivity (*r* = −0.07, *p* < 0.05). However, the small effect sizes suggest limited practical significance despite statistical significance, likely influenced by the large sample size. No significant associations were observed between age and FoMO or poorer sleep quality (Table 3).

**Table 3:** Correlations among study variables (*N* = 1,071).

Variable	1	2	3	4	5
1. FoMO score	1				
2. Problematic social media use	0.550^**^	1			
3. Academic productivity	0.072^*^	−0.114^**^	1		
4. Sleep quality	0.248^**^	0.202^**^	0.240^**^	1	
5. Age	0.037	0.079^**^	−0.067^*^	−0.005	1

Values are Pearson correlation coefficients (*r*). ^*^p < 0.05, ^**^p < 0.01.

### Academic productivity

Multiple linear regression analysis was conducted to examine the association between variables and academic productivity. The overall model was statistically significant (*F* = 19.40, *p* < 0.001), explaining approximately 10% of the variance in academic productivity (*R*^2^ = 0.10). Although statistically significant, the relatively low *R*^2^ value indicates that most of the variance in academic productivity remains unexplained, suggesting that additional psychological, academic, and environmental factors may contribute to this outcome. FoMO was positively associated with academic productivity (*β* = 0.16, *p* < 0.001), whereas problematic social media use was negatively associated with academic productivity (*β* = −0.17, *p* < 0.001). Study hours showed the strongest positive association with academic productivity (*β* = 0.19, *p* < 0.001), while GPA was also positively associated with academic productivity (*β* = 0.11, *p* < 0.001). Gender and age were not significantly associated with academic productivity (*p* > 0.05) (Table 4).

**Table 4:** Regression analysis of variables associated with academic productivity.

Predictor	*B*	95% CI		SE	*β*	*p*-value
		Lower	Upper			
FoMO	0.19	0.11	0.27	0.04	0.16	<0.01
Problematic social media use	−0.19	−0.26	−0.11	0.04	−0.17	<0.01
Gender	0.11	−0.01	0.23	0.06	0.05	0.08
Age	−0.02	−0.03	0.01	0.04	−0.02	0.52
Study hours	0.12	0.08	0.16	0.02	0.19	<0.01
GPA	0.10	0.04	0.15	0.03	0.11	<0.01

Model fit: *R*^2^ = 0.10, *F* = 19.40, *p* < 0.001.

### Poorer sleep quality

Multiple linear regression analysis was performed to examine the associations between variables and poorer sleep quality. The model was statistically significant (*F* = 22.06, *p* < 0.001), explaining approximately 11% of the variance in sleep quality (*R*^2^ = 0.11). However, the relatively low *R*^2^ value suggests that additional behavioral, psychological, and lifestyle factors may contribute to poorer sleep quality among university students. FoMO was positively associated with poorer sleep quality (*β* = 0.19, *p* < 0.001), and problematic social media use was also positively associated with poorer sleep quality (*β* = 0.11, *p* = 0.002). Gender was significantly associated with poorer sleep quality (*β* = 0.11, *p* < 0.001), indicating differences between male and female participants. Study hours were positively associated with sleep quality (*β* = 0.15, *p* < 0.001), whereas daily social media use and age were not significantly associated with poorer sleep quality (*p* > 0.05) (Table 5).

**Table 5:** Regression analysis of variables associated with poorer sleep quality.

Predictor	*B*	95% CI		SE	*β*	*p*-value
		Lower	Upper			
FoMO	0.20	0.12	0.27	0.04	0.19	<0.001
Problematic social media use	0.12	0.06	0.21	0.04	0.11	0.002
Gender	0.20	0.09	0.31	0.06	0.11	<0.001
Age	−0.00	−0.03	0.02	0.03	−0.00	0.90
Study hours	0.09	0.05	0.12	0.02	0.15	<0.001
Daily social media use	0.04	−0.00	0.09	0.02	0.06	0.06

Model fit: *R*^2^ = 0.11, *F* = 22.06, *p* < 0.001.

## Discussion

The present study examined the relationships among fear of missing out (FoMO), problematic social media use, academic productivity, and sleep quality among university students in Saudi Arabia. Overall, the findings were generally consistent with the proposed conceptual framework and highlighted the interconnected role of psychological and behavioral factors in student well-being and academic functioning. Consistent with previous research, FoMO was positively associated with problematic social media use (Gori et al., 2023; Rozgonjuk et al., 2020; Franchina et al., 2018; Akbari et al., 2021). This finding supports the conceptualization of FoMO as a psychological factor that may encourage persistent online engagement and compulsive digital behaviors (Elhai et al., 2016). Within the framework of Self-Determination Theory (SDT), FoMO may reflect unmet relatedness needs that motivate individuals to seek social connectedness through digital platforms (Przybylski et al., 2013). The observed relationship suggests that students experiencing higher FoMO may engage more frequently with social media to maintain social awareness and avoid perceived exclusion (Wegmann et al., 2017).

Problematic social media use was negatively associated with academic productivity, consistent with prior studies linking excessive digital engagement with distraction, procrastination, reduced concentration, and impaired academic performance (Sun and Chao, 2024; Barton et al., 2018). Excessive social media use may interfere with effective study habits and time management, thereby reducing students’ ability to maintain consistent academic engagement (Kirschner and Karpinski, 2010).

Interestingly, FoMO showed a weak positive correlation with academic productivity. This association may suggest that some students experiencing FoMO remain academically engaged to maintain competitiveness, social relevance, or peer connectedness within academic settings (Alt, 2015; Riordan et al., 2015). From an SDT perspective, students with higher FoMO may sustain academic engagement as a means of preserving social belonging and participation within peer network (Przybylski et al., 2013). These findings highlight the potentially complex role of FoMO in influencing both maladaptive and achievement-oriented behaviors among university students.

Both FoMO and problematic social media use were associated with poorer sleep quality. These findings are consistent with previous literature suggesting that excessive nighttime social media engagement and emotional attachment to online interactions may contribute to delayed sleep onset and sleep disturbances (Almeida et al., 2023; Khan et al., 2024). FoMO may also increase psychological arousal and the urge to remain continuously connected, particularly during nighttime hours, thereby interfering with healthy sleep behaviors (Almeida et al., 2023). Academic productivity was also positively associated with poorer sleep quality. Although the association was statistically significant, the effect size was weak and should therefore be interpreted cautiously. This finding may reflect increased academic demands, prolonged study hours, academic stress, and irregular sleep schedules among highly academically engaged students (Creswell et al., 2023). Together, these findings emphasize the multidimensional relationship between digital behaviors, academic demands, and sleep health among university students.

The analysis of demographic variables showed limited differences in FoMO across participant characteristics. Significant differences were observed across academic disciplines, living arrangements, and duration of social media use, whereas gender, age, GPA, and academic year were not significantly associated with FoMO. Students in health-related disciplines and those living independently reported higher FoMO levels, which may reflect differences in academic pressure, social interaction patterns, or lifestyle demands. These findings are consistent with previous studies indicating that FoMO is more strongly associated with psychosocial and behavioral factors than with demographic characteristics alone (Thomas and George, 2025; Nedim Bal et al., 2025; Piko et al., 2025).

### Implications

The findings of this study have important implications. First, they emphasize the need for interventions that address FoMO and problematic social media use among university students. Educational programs that promote digital well-being, time management, and healthy technology habits may help reduce the negative effects of excessive social media use (Carras et al., 2024; Wahdiyati et al., 2023). Second, the observed relationships suggest that addressing social media behaviors may improve both academic productivity and sleep quality. Universities may consider integrating awareness campaigns or workshops focusing on responsible digital use and sleep hygiene (Lu et al., 2024). Finally, the results emphasize the importance of considering both psychological factors (FoMO) and behavioral factors (social media use) when examining student well-being and academic outcomes (Thomas and George, 2025).

### Limitations and future research

Several limitations should be considered when interpreting the findings of this study. First, the cross-sectional design limits the ability to establish causal relationships or determine the directionality of the observed associations among FoMO, problematic social media use, academic productivity, and poorer sleep quality. Second, all data were collected using self-reported measures, which may increase the risk of reporting bias, social desirability bias, and common method bias. Although a formal statistical assessment of common method bias, such as Harman’s single-factor test, was not conducted, several procedural approaches were used to reduce its potential impact, including anonymous data collection, voluntary participation, and the use of standardized validated measures. In addition, the relatively short test–retest interval used during pilot testing may not fully capture the temporal stability of the study measures and may have increased the possibility of recall bias.

The use of convenience and snowball sampling may have introduced selection bias and limited the representativeness and generalizability of the findings. The predominance of female participants may also have influenced the observed results, although this distribution generally reflects female enrollment patterns within Saudi universities. Furthermore, the regression models explained only a modest proportion of the variance in academic productivity and poorer sleep quality, suggesting that these outcomes are influenced by multiple interconnected psychological, behavioral, academic, and environmental factors beyond FoMO and problematic social media use. Additional unmeasured variables, including mental health status, personality traits, academic stress, coping strategies, sleep habits, and environmental influences, may also contribute to these outcomes. Moreover, some statistically significant associations, particularly the positive association between FoMO and academic productivity, demonstrated weak correlation coefficients and small effect sizes and should therefore be interpreted cautiously despite statistical significance.

Despite these limitations, the study included a large and diverse sample drawn from multiple universities and academic disciplines across Saudi Arabia, enhancing the contextual relevance and exploratory value of the findings. Future research should employ probability-based sampling methods, longitudinal designs, and objective measures of digital behavior and sleep patterns to better examine causal pathways and strengthen external validity. Future studies should also consider mediation and moderation analyses to provide a more comprehensive understanding of the complex relationships among psychological, behavioral, academic, and sleep-related factors. Continued investigation of these multidimensional pathways may further clarify the psychological and behavioral mechanisms underlying academic functioning and sleep health among university students.

## Conclusion

FoMO is associated with increased social media use and poorer sleep quality, while problematic social media use was negatively associated with academic productivity. These findings highlight the complex interplay between psychological and behavioral factors in shaping student outcomes. Addressing FoMO alongside promoting balanced digital habits and healthier sleep practices may be associated with improved well-being. Future research should further explore underlying mechanisms and causal relationships using longitudinal approaches.

## Data Availability

The original contributions presented in the study are included in the article/supplementary material, further inquiries can be directed to the corresponding author.
